# Effects of abdominal aortic aneurysm on long-term survival in lung cancer patients

**DOI:** 10.1038/s41598-023-46196-8

**Published:** 2024-01-08

**Authors:** Hyangkyoung Kim, Tae-Won Kwon, Yong-Pil Cho, Jun Gyo Gwon, Youngjin Han, Sang Ah Lee, Ye-Jee Kim, Seonok Kim

**Affiliations:** 1 Department of Surgery, College of Medicine, Ewha Womans University, Seoul, Korea; 2 Division of Vascular Surgery, Department of Surgery, College of Medicine and Asan Medical Center, University of Ulsan, Seoul, Korea; 3 Department of Emergency Critical Care Trauma Surgery, Korea University Guro Hospital, 148 Gurodong-ro Guro-gu, Seoul, 08308 Korea; 4 Armed Forces Trauma Center, Armed Forces Capital Hospital, Songnam, Korea; 5 Department of Clinical Epidemiology and Biostatistics, Asan Medical Center, University of Ulsan College of Medicine, Seoul, Korea

**Keywords:** Diseases, Oncology

## Abstract

The major causes of death in patients with abdominal aortic aneurysm (AAA) are cardiovascular disease and cancer. The purpose of this study was to evaluate the effect of AAA on long-term survival in lung cancer patients. All patient data with degenerative type AAA and lung cancer over 50 years of age during the period 2009 to 2018 was collected retrospectively from a National Health Insurance Service (NHIS) administrative database and matched to lung cancer patients without AAA by age, sex, metastasis, and other comorbidities. Mortality rate was compared between the groups. A total of 956 AAA patients who could be matched with patients without AAA were included, and 3824 patients in the matched group were used for comparison. Patients with AAA showed higher risk of death compared with the matched cohort (adjusted hazard ratio (HR) 1.14, 95% confidence interval (CI) 1.06–1.23, *p* < 0.001). When compared to a matched group of untreated AAA patients, patients with of history of AAA exhibited a significantly increased risk of overall mortality [HR (95%CI) 1.219 (1.113–1.335), *p* < .001, adjusted HR (95% CI) 1.177 (1.073–1.291), *p* = .001]. By contrast, mortality risk of AAA patients treated either by endovascular abdominal aortic repair or open surgical repair was not significantly different from that of the matched group (*p* = 0.079 and *p* = 0.625, respectively). The mortality risk was significantly higher when AAA was present in lung cancer patients, especially in patients with unrepaired AAA, suggesting the need for continuous cardiovascular risk management.

## Introduction

Abdominal aortic aneurysm (AAA) is a localized enlargement of the abdominal aorta, where the diameter exceeds 3 cm or is more than 50% larger than normal^1^. Traditionally, AAAs were recognised as being associated with rupture risk, and efforts were focused on early diagnosis and minimising morbidity and mortality related to repair^2^. The majority of deaths during long-term follow-up after AAA repair are non-aneurysm-related causes, and the predominant causes of death in AAA patients are cardiovascular and cancer-related^3^. There has been an increase in reports of concomitant AAA and malignancy, and an estimated 1.0–17.0% of patients with AAA have or develop a concomitant malignancy^4,5^. Both AAA and malignancy have a tendency to increase in prevalence with age^6,7^. In addition to advanced age, coexistence of these diseases may be attributable to similar patient demographics and common risk factors such as smoking^8^. Our previous study evaluated the association of AAA with cancer, and we found that the prevalence of AAA in cancer patients was even higher than that of heart failure, which is widely known to be highly associated with AAA^9,10^. The effect of the coexistence of the two diseases on the survival of patients has attracted considerable interest, and there have been studies on the effects of cancer in AAA patients. The progression of small AAAs does not appear to be significantly affected by cancer or chemotherapy^11,12^. As cancer is the main cause of death in patients with AAA repair, however, a higher mortality rate is expected in patients with a cancer history, and this has been shown to significantly worsen long-term outcomes after endovascular abdominal aortic repair (EVAR) or open surgical repair (OSR)^13,14^. By contrast, the effect of AAA on survival in cancer patients has not been evaluated, and this association in assessed in the present study.

## Methods

All data of AAA patients over 50 years of age in a National Health Insurance Service (NHIS) administrative database covering approximately 98% of the South Korean population was collected retrospectively for the period 2009 to 2018. The study was conducted in accordance with the guidelines of the Declaration of Helsinki and was approved by Asan medical center Institutional Review Board (approval number: 2020-1242) and informed written consent was waived by Asan medical center Institutional Review Board due to the retrospective nature of the study. The manuscript follows the guidelines of the RECORD statement, extended from the STROBE statement.

**Figure 1:**
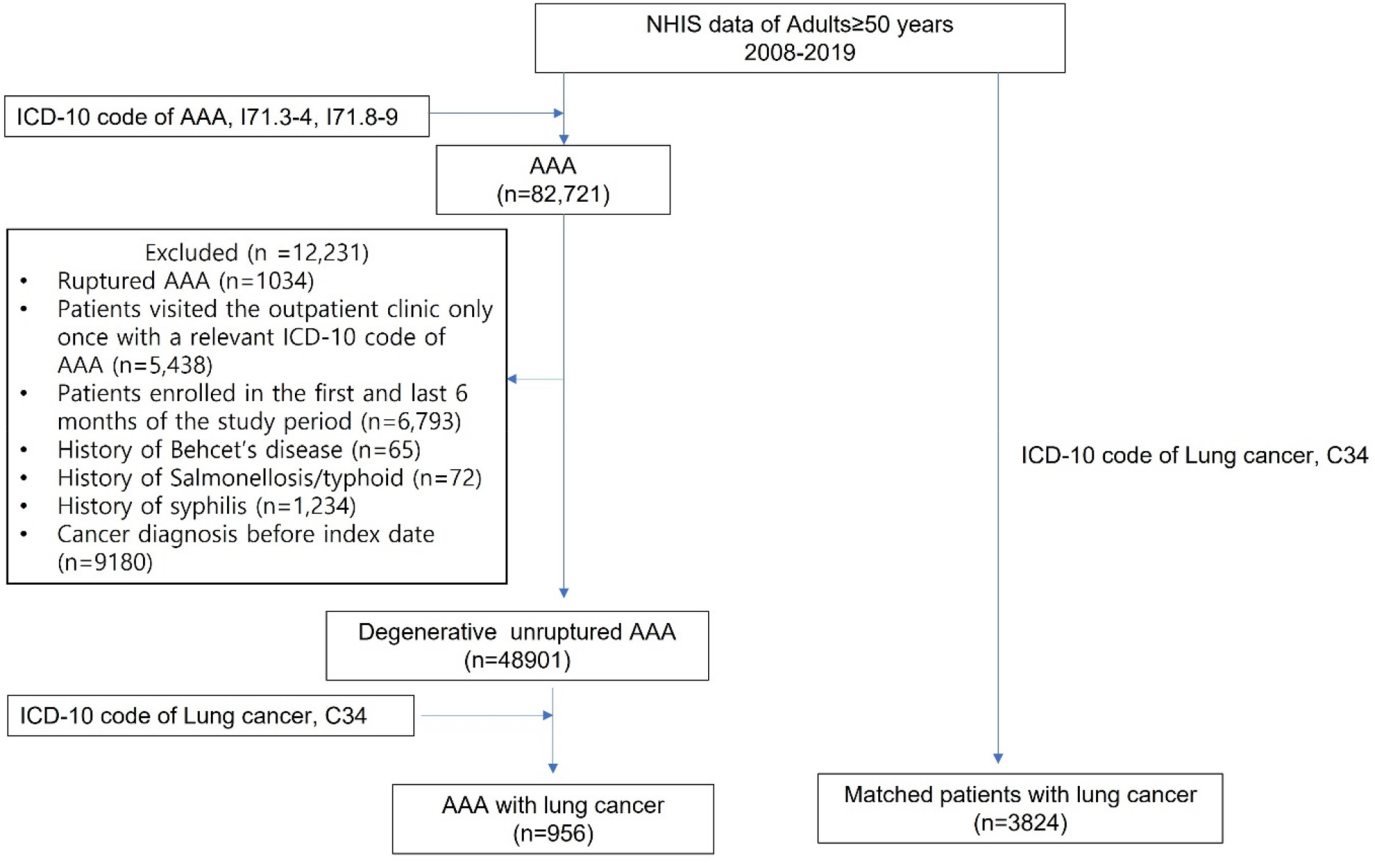
Flow diagram.

The inclusion and exclusion criteria for AAA are as follows: Patients over 50 years with a degenerative type of AAA are included, while ruptured AAA and non-degenerative types of AAA are excluded. A flow diagram illustrating the selection of study participants is depicted in Fig. 1. To exclude non-degenerative cases of AAA, such as those related to trauma, only the data of patients over 50 years of age was accessed. Patients with AAA were identified by ICD-10 codes including I71.3–4 and I71.8–9. Initial screening included ruptured AAA cases because the diagnosis of some cases was miscoded. Patients with a rupture code who survived more than 90 days without treatment were classified as unruptured AAA. Ruptured AAA patients were then excluded. Patients with a relevant ICD-10 code of AAA who visited the outpatient clinic only once were excluded due to uncertainty of the diagnosis. In order to limit the analysis to degenerative AAA, patients with the following conditions were excluded: (1) AAA related to Behcet’s disease (ICD-10 code M35.2) or syphilis (ICD-10 code A50-53), and (2) history of typhoid fever or salmonellosis (ICD-10 code A02, O2034, O2036, O2039) within 6 months of AAA diagnosis. Patients who were enrolled during the first and last 6 months of the study period and patients who were diagnosed with cancer before the index date were also excluded. The AAA index date was defined as the date of the first AAA diagnosis. Patients with a history of cancer were defined as those diagnosed with lung cancer (ICD-10 C34) more than two times after the AAA index date. Then the control group was sampled from among lung cancer patients who had not been diagnosed with AAA during the same period after matching for age, sex, and metastasis (1:4 matching). The main outcome was the rate of all-cause mortality for the AAA group and the matched lung cancer group.

### Variables of interest

The demographic variables included age, sex, and comorbidities. The comorbidities were selected using the Charlson Comorbidity Index (CCI), a method of categorising comorbidities based on ICD-10 codes, and grouped based on CCI scores 0-1, 2, and ≥ 3^12^. Specific comorbidities included hypertension (ICD-10: I10), diabetes mellitus (ICD-10: E10, E11), myocardial infarction (ICD-10: I21, I22), and end-stage renal diseases (ICD-10: N18.5).

### Statistical analysis

The statistical differences in the patient characteristics based on the history of AAA were computed using the standardised mean difference (SMD), with a SMD of less than 0.1 considered to indicate a balance between the groups. The prevalence of all-cause mortality in cancer patients with and without a history of AAA was compared. All-cause mortality was calculated at 30 days and at the end of the study period after AAA repair. The overall mortality rate is presented along with the age-standardised rate. Kaplan–Meier curves was used to depict the cumulative incidence of all-cause mortality. A statistical comparison between the survival of the AAA group and that of the matched group was performed to estimate the effect of AAA on death in cancer patients treated by EVAR or OSR. Univariate (crude) and multivariate (adjusted) Cox-proportional hazards regression analyses were performed to assess associations between AAA and the occurrence of all-cause deaths. Hazard ratios (HRs) and 95% confidence intervals (CIs) for the outcomes were calculated in AAA patients with and without a cancer history. The Cox-proportional regression models were adjusted for CCI. All analyses were performed using SAS 7.1 software (SAS Institute, Cary, NC, USA), and the statistical significance level was set to α = 0.05.

## Results

Among patients with degenerative unruptured AAA, 4984 patients had cancer, and the most common types were lung (20.0%), stomach (11.9%), prostate (8.3%), and colon (7.1%). Among 995 lung cancer patients, 956 patients who could be matched with those without AAA were used for the analysis, and 3824 patients in the matched group were used for comparison.

Patients’ characteristics are summarised in Table 1. There were no significant differences in the matched variables (sex, age and metastasis) between the two groups (SMD < 0.001). With the exception of diabetes mellitus, patients with cancer had more comorbidities (SMD = 0.013) and a higher CCI score (SMD = 0.274).

**Table 1:** Patients’ characteristics.

	AAA with cancer	Matched cohort	SMD
	(n = 956)	(*n* = 3824)	
Age (years), mean (SD)	75.0 (7.0)	75.0 (7.0)	< .001
Men, n (%)	832 (87.0)	3328 (87.0)	< .001
Comorbidities, n (%)			
Diabetes mellitus	329 (34.4)	1340 (35.0)	0.013
Hypertension	739 (77.3)	2356 (61.6)	0.346
Dyslipidemia	708 (74.1)	2068 (54.1)	0.426
Chronic kidney disease	100 (10.5)	144 (3.8)	0.263
ESRD	19 (2.0)	19 (0.5)	0.135
Cerebrovascular accident	8 (0.8)	24 (0.6)	0.177
Ischaemic heart disease	35 (3.7)	39 (1.0)	0.17
Heart failure	186 (19.5)	379 (9.9)	0.272
Charlson Comorbidity Index, mean (SD)	5.81 (3.64)	4.95 (3.53)	0.24
0–2	166 (17.4)	1021 (26.7)	0.274
3–4	241 (25.2)	1083 (28.3)	
5	549 (57.4)	1720 (45.0)	
Alcohol, n (%)			0.149
None	455 (47.6)	1697 (44.4)	
< 3 d/week	152 (15.9)	737 (19.3)	
≥ 3 d/week	46 (4.8)	282 (7.4)	
No response/Missing	303 (31.7)	1108 (28.9)	
Smoking, n (%)			0.151
Non-smoker	194 (20.3)	950 (24.8)	
Ex-smoker	215 (22.5)	958 (25.1)	
Current smoker	244 (25.5)	810 (21.2)	
No response/Missing	303 (31.7)	1106 (28.9)	
Cancer surgery, n (%)	772 (80.8)	3008 (78.7)	0.052
Chemotherapy, n (%)	81 (8.5)	285 (7.5)	0.038

SMD, standardised mean difference.

**Figure 2:**
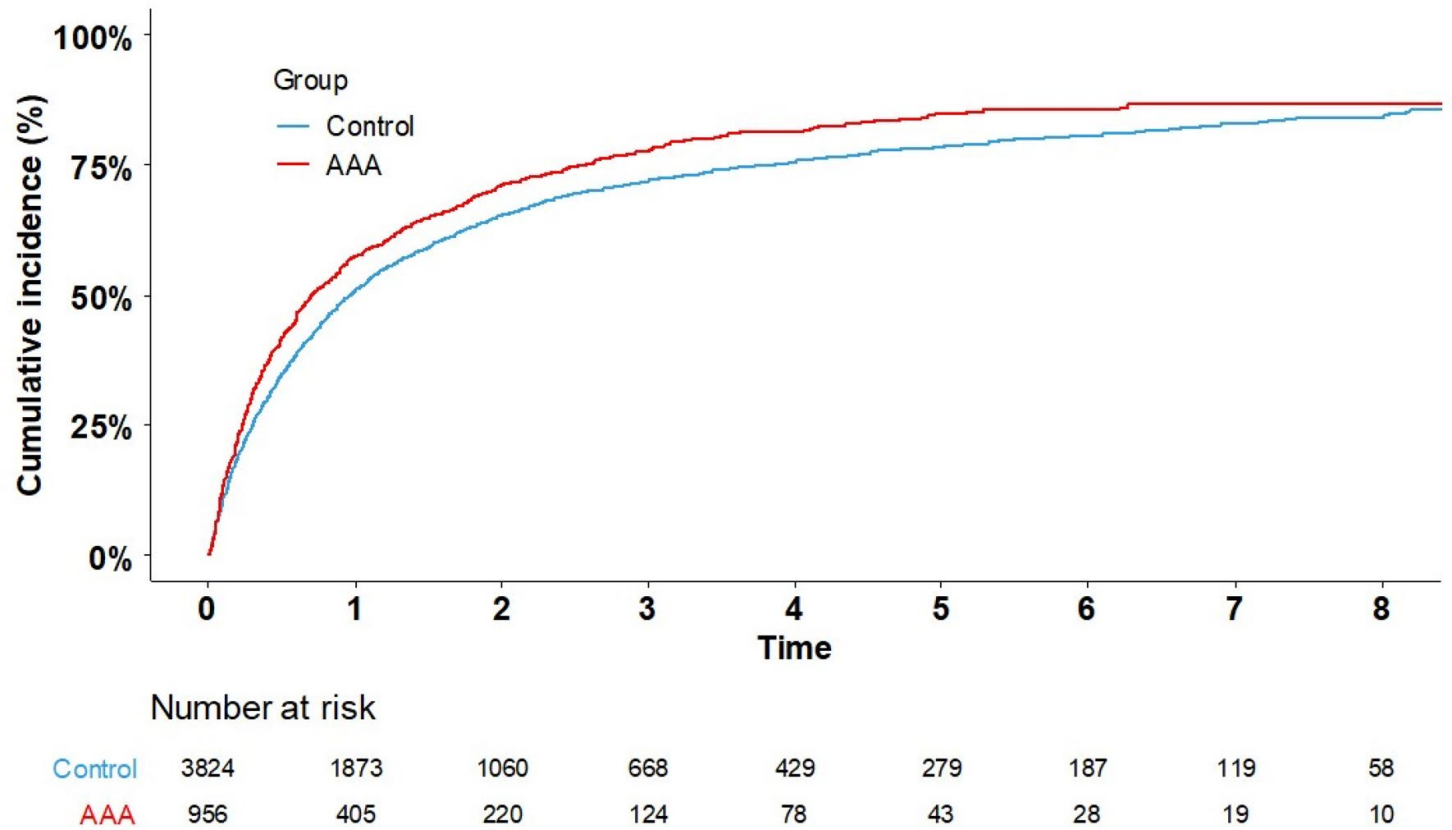
Risk of death of lung cancer patients with abdominal aortic aneurysm (AAA) and of the matched control.

Figure 2 shows Kaplan–Meier curves for the cumulative incidence of overall mortality for patients with a history of AAA. The median follow-up period was 6.13 years (interquartile range: 3.05–6.54). AAA patients with lung cancer had a higher overall mortality risk than those in the matched non-AAA group. During the entire study period, the risk of death among patients with AAA was high, and both curves converged to approximately 75% after 8 years. Table 2 shows that the all-cause mortality rate in lung cancer patients with AAA and the matched controls. Patients with AAA had a higher risk of death than the matched cohort (adjusted HR 1.14, 95% CI 1.06–1.23, *p* < 0.001). The risk of death was compared between the patients with AAA and matched cohorts based on the variables used for matching, including age, sex, and metastasis. In men, patients in the AAA group had a higher risk of death than those in the matched group, even after adjustment for CCI (adjusted HR 1.16, 95% CI 1.07–1.26, *p* < 0.001). By contrast, in women, there were no significant differences between the two groups (*p* > 0.05). AAA patients who were diagnosed with lung cancer at ages 65–79 or > 80 years had a higher risk of death than the controls (*p* = 0.005 and *p* = 0.021, respectively). Patients with AAA had a higher risk of death irrespective of whether they had metastasis or not (*p* = 0.039 and *p* = 0.001, respectively).

**Table 2:** Comparison of mortality rate between lung cancer patients with abdominal aortic aneurysm (AAA) and matched control (no AAA).

	AAA (n = 956)				no AAA (n = 3824)				HR (95% CI)	P	HR^a^ (95% CI)	P
	Total	Death	PY	Mortality rate (/100PYs)	Total	Death	PY	Mortality rate (/100PYs)				
Total	956	747	1303.8	57.3	3824	2815	6288.1	44.8	1.19 (1.10–1.28)	< .001	1.14 (1.06–1.23)	0.001
Sex												
Men	832	661	1090.8	60.6	3328	2488	5360.9	46.4	1.20 (1.11–1.30)	0.000	1.16 (1.07–1.26)	< 0.001
Women	124	86	213.0	40.4	496	327	927.1	35.3	1.11 (0.90–1.38)	0.341	1.05 (0.84–1.30)	0.690
Age at Dx*												
50–64	81	47	200.3	23.5	324	166	853.2	19.5	1.18 (0.91–1.54)	0.216	1.08 (0.82–1.43)	0.573
65–79	625	479	906.0	52.9	2500	1815	4394.3	41.3	1.19 (1.08–1.32)	0.000	1.16 (1.04–1.28)	0.005
80 +	250	221	197.6	111.9	1000	834	1040.6	80.1	1.22 (1.06–1.40)	0.006	1.18 (1.03–1.37)	0.021
Metastasis												
No	829	630	1211.2	52.0	3316	2365	5813.1	40.7	1.19 (1.09–1.29)	< .001	1.16 (1.06–1.26)	0.001
Yes	127	117	92.6	126.3	508	450	475.0	94.7	1.24 (1.02–1.51)	0.0324	1.24 (1.01–1.51)	0.039

*Age at lung cancer diagnosis.

HR calculated to consider matched nature.

HR^a^ adjusted for Charlson Comorbidity Index.

PY, person years; HR, hazard ratio; CI, confidence interval; Dx, diagnosis.

**Figure 3:**
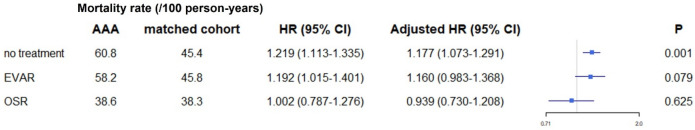
Forest plot comparing the mortality rate in patients with abdominal aortic aneurysm (AAA) and their matched cohort using treatment-related variables, including no treatment, endovascular aneurysm repair (EVAR), and open surgical repair (OSR). Adjusted HR; adjusted for Charlson Comorbidity Index.

In the AAA group, 416 patients underwent treatment for AAA; 232 with endovascular abdominal aortic aneurysm repair (EVAR) and 84 with open surgical repair (OSR). Demographic data of subgroups are summarised in Supplementary Table 1. Figure 3 depicts the HRs for the mortality rate of AAA and matched group patients grouped by treatment type. When the matched group was compared with the untreated AAA patients, patients with a history of AAA exhibited a significantly higher risk of overall mortality [HR (95%CI) 1.219 (1.113–1.335), *p* < 0.001, adjusted HR (95% CI) 1.177 (1.073–1.291), *p* = 0.001] than those without. Conversely, the mortality risk of AAA patients treated either by EVAR or OSR was not significantly different from the matched group (*p* = 0.079 and *p* = 0.625, respectively).

## Discussion

The long-term prognosis in patients with AAA has been reported in many studies^15,16^. Despite improvements in short-term outcome after AAA repair, late survival has not improved, even after successful repair^17,18^. Previous studies have suggested a high prevalence of AAA in patients with lung cancer^6,19^. It is not clearly demonstrated whether this is a simple co-occurrence or if there is an association between the two diseases^20^. However, it is suggested that similar patient demographics, such as advanced age, male sex, and smoking, and a common pathway of oxidative stress, can potentially play a role in the co-existence of these diseases^21,22^. As cancer is suggested to be one of the major causes of death, we investigated how AAA affects survival in patients with lung cancer. In this study, we found that the presence of AAA negatively affected long-term survival. When the cohort was grouped by type of treatment, a worse outcome was observed in patients who did not receive treatment, and there was no significant difference observed between patients who had undergone EVAR or OSR. Because we eliminated ruptured cases, there was no repair in the untreated group during the study period, and those patients were presumed to have small AAAs that did not require treatment. Therefore, this group included AAA patients who were free of surgery-related morbidity and rupture-related death. It is intriguing that the mortality risk was increased in this group but not in the treated group. A firm conclusion, however, cannot be drawn about whether AAA itself raises the mortality risk. Rather, it seems more reasonable to assume that confounding factors associated with AAA contribute to the mortality risk. In our cohort, cardiovascular comorbidities, including hypertension, dyslipidemia, chronic kidney disease, end-stage renal disease, and ischaemic heart disease, were more prevalent in AAA patients. Previous reports suggested that advanced atherosclerosis or cardiovascular disease affected long-term survival of AAA patients^23,24^. Therefore, there would appear to be a need for mandatory cardiovascular risk management during follow-up of patients with small AAAs and cancer to improve long-term outcomes.

By contrast, patients who underwent either EVAR or OSR exhibited a mortality risk similar to that of the control group. It seems rather strange that there was no difference in mortality risk between the matched groups of patients with large AAAs that were indicated for treatment. After successful surgical repair of an abdominal aortic aneurysm, patients have an increased risk of death from cardiovascular causes for many years, and a significant association between aortic diameter and cardiovascular mortality has been reported^25^. However, the EVAR group included more patients with comorbidities, and patients who underwent EVAR had a higher mortality risk in the unadjusted model. Meanwhile, patients who underwent OSR and the matched group had a lower mortality rate. It seems possible that patients with OSR could have been selected because of a low surgical risk.

The principal limitations of the present study are its retrospective design and the uncertainty in the assignment of cancer stage due to the nature of the administrative database. Furthermore, potential confounding variables, including smoking status, comorbidities, and socioeconomic factors, may have influenced the mortality outcomes, which were not comprehensively controlled for in our analysis. The smoking habit appeared to have the most significant influence on mortality outcomes among them, and an effort was made to incorporate this information into the analysis by integrating national health check-up data with our database. However, approximately 30% of the data was encountered being missing. However, we tried to overcome this limitation by matching metastatic status when defining the control group. The strength of the present study includes its size and long-term follow-up without censoring. The present study also benefited from the inclusion of patients who were diagnosed with cancer after the diagnosis of AAA, which reduced bias from the morbidity period of cancer. Another strength of this study is that it evaluated survival in untreated patients.

The effect of AAA on survival of lung cancer patients reported here may have implications for management strategies of AAA patients regardless of treatment in the future. The ultimate goal is to reduce cardiovascular risk and improve long-term survival in this population. Future studies conducted in international settings or incorporating a comprehensive assessment of cancer staging and different treatment modalities are necessary to enhance the universal applicability of our findings. Furthermore, given the significance of gender differences in AAA and lung cancer, it is crucial to prioritize gender diversity in their participant selection.

## Conclusions

The significant increase in mortality of lung cancer patients with AAA, especially untreated patients, suggests that cardiovascular risk should be managed to improve long-term survival of cancer patients.

### Supplementary Information

Supplementary Table 1.

## Data Availability

The data that support the findings of this study are available from National Health Insurance Service of Korea but restrictions apply to the availability of these data, which were used under license for the current study, and so are not publicly available. Data are however available from the corresponding author upon reasonable request and with permission of National Health Insurance Service of Korea.
