# Supplementary material for: Effects of abdominal aortic aneurysm on long-term survival in lung cancer patients
Source: Sci Rep. 2024 Jan 8;14:781. doi: 10.1038/s41598-023-46196-8 (PMC10774350; doi:10.1038/s41598-023-46196-8)
Supplement: Supplementary file 1 — Supplementary Table 1. [file 41598_2023_46196_MOESM1_ESM.docx]

|  | **AAA (*n*=640)** | | **Matched cohort (*n*=2560)** | | **SMD** |
| --- | --- | --- | --- | --- | --- |
| **Age** [mean (standard deviation)] | 74.98 | (7.25) | 74.95 | (7.18) | 0.004 |
| **Men** (*n,* %) | 537 | (83.9) | 2148 | (83.9) | <0.001 |
| **Metastasis** (*n,* %) | 96 | (15.0) | 384 | (15.0) | <0.001 |
| **Comorbidities** (*n,* %) |  |  |  |  |  |
| Diabetes mellitus | 221 | (34.5) | 881 | (34.4) | 0.002 |
| Hypertension | 483 | (75.5) | 1574 | (61.5) | 0.304 |
| Dyslipidemia | 448 | (70.0) | 1363 | (53.2) | 0.350 |
| Chronic kidney disease | 50 | (7.8) | 92 | (3.6) | 0.183 |
| End stage kidney disease | 9 | (1.4) | 14 | (0.5) | 0.087 |
| Cerebrovascular disease | 143 | (22.3) | 380 | (14.8) | 0.194 |
| Ischemic heart disease | 33 | (5.2) | 57 | (2.2) | 0.156 |
| Heart failure | 121 | (18.9) | 250 | (9.8) | 0.263 |
| Charson Cormorbidity Index [mean (SD)] | 5.82 | 3.73 | 5.03 | 3.62 | 0.215 |
| 0,1,2 | 115 | (18.0) | 684 | (26.7) | 0.247 |
| 3,4 | 161 | (25.2) | 703 | (27.5) |  |
| 5+ | 364 | (56.9) | 1173 | (45.8) |  |
| **Smoking** (*n,* %) |  |  |  |  |  |
| No response | 2 | (0.3) | 6 | (0.2) | 0.143 |
| Never smoker | 144 | (22.5) | 668 | (26.1) |  |
| Previous smoker | 135 | (21.1) | 630 | (24.6) |  |
| Current smoker | 153 | (23.9) | 527 | (20.6) |  |
| Not available | 206 | (32.2) | 729 | (28.5) |  |
| **Alcohol use** (*n,* %) |  |  |  |  |  |
| No response | 2 | (0.3) | 6 | (0.2) | 0.136 |
| None | 299 | (46.7) | 1155 | (45.1) |  |
| Mild-to-moderate | 99 | (15.5) | 483 | (18.9) |  |
| Heavy | 34 | (5.3) | 187 | (7.3) |  |
| Not available | 206 | (32.2) | 729 | (28.5) |  |
| **Cancer surgery** (*n,* %) | 123 | (19.2) | 530 | (20.7) | 0.037 |
| **Chemotherapy** (*n,* %) | 586 | (91.6) | 2358 | (92.1) | 0.020 |

**Supplementary Table 1**. Demographic data of subgroups. A. untreated abdominal aortic aneurysm (AAA) patients and their matched cohort; B. AAA patients treated with endovascular aneurysm repair and their matched cohort; C. AAA patients treated with open surgical repair and their matched cohort. SMD, standardised mean difference

A.

B.

|  | | **AAA (n=232)** | | | | **Matched cohort (n=928)** | | **SMD** |
| --- | --- | --- | --- | --- | --- | --- | --- | --- |
| **Age** [mean (standard deviation, SD)] | | 75.47 | | 6.61 | | 75.43 | 6.57 | 0.007 |
| **Men** (*n,* %) | | 215 | | (92.7) | | 860 | (92.7) | <.001 |
| **Metastasis** (*n,* %) | | 20 | | (8.6) | | 80 | (8.6) | <.001 |
| **Comorbidities** (*n,* %) | |  | |  | |  |  |  |
| Diabetes mellitus | | 77 | | (33.2) | | 345 | (37.2) | 0.084 |
| Hypertension | | 188 | | (81.0) | | 573 | (61.7) | 0.437 |
| Dyslipidemia | | 189 | | (81.5) | | 535 | (57.7) | 0.536 |
| Chronic kidney disease | | 38 | | (16.4) | | 40 | (4.3) | 0.404 |
| End stage kidney disease | | 7 | | (3.0) | | 2 | (0.2) | 0.224 |
| Cerebrovascular disease | | 50 | | (21.6) | | 137 | (14.8) | 0.177 |
| Ischemic heart disease | | 15 | | (6.5) | | 21 | (2.3) | 0.207 |
| Heart failure | | 49 | | (21.1) | | 102 | (11.0) | 0.279 |
| Charson Cormorbidity Index [mean (SD)] | 5.73 | | (3.38) | | 4.79 | | (3.30) | 0.281 |
| 0,1,2 | | 37 | | (15.9) | | 246 | (26.5) | 0.323 |
| 3,4 | | 57 | | (24.6) | | 268 | (28.9) |  |
| 5+ | | 138 | | (59.5) | | 414 | (44.6) |  |
| **Smoking** (*n,* %) | |  | |  | |  |  |  |
| No response | |  | |  | |  |  |  |
| Never smoker | | 39 | | (16.8) | | 222 | (23.9) | 0.240 |
| Previous smoker | | 61 | | (26.3) | | 233 | (25.1) |  |
| Current smoker | | 64 | | (27.6) | | 200 | (21.6) |  |
| Not available | | 68 | | (29.3) | | 273 | (29.4) |  |
| **Alcohol use** (*n,* %) | |  | |  | |  |  |  |
| No response | | 0 | | (0.0) | | 2 | (0.2) | 0.258 |
| None | | 115 | | (49.6) | | 389 | (41.9) |  |
| Mild-to-moderate | | 39 | | (16.8) | | 190 | (20.5) |  |
| Heavy | | 10 | | (4.3) | | 74 | (8.0) |  |
| Not available | | 68 | | (29.3) | | 273 | (29.4) |  |
| **Cancer surgery** (*n,* %) | | 44 | | (19.0) | | 207 | (22.3) | 0.083 |
| **Chemotherapy** (*n,* %) | | 214 | | (92.2) | | 872 | (94.0) | 0.068 |

C.

|  | **AAA (*n*=84)** | | **Matched cohort (*n*=336)** | | **SMD** |
| --- | --- | --- | --- | --- | --- |
| **Age** [mean (standard deviation)] | 73.81 | (6.44) | 73.8 | (6.39) | 0.001 |
| **Men** (*n,* %) | 80 | (95.2) | 320 | (95.2) | <.001 |
| **Metastasis** (*n,* %) | 11 | (13.1) | 44 | (13.1) | <.001 |
| **Comorbidities** (*n,* %) |  |  |  |  |  |
| Diabetes mellitus | 31 | (36.9) | 114 | (33.9) | 0.062 |
| Hypertension | 68 | (81.0) | 209 | (62.2) | 0.425 |
| Dyslipidemia | 71 | (84.5) | 170 | (50.6) | 0.778 |
| Chronic kidney disease | 12 | (14.3) | 12 | (3.6) | 0.383 |
| End stage kidney disease | 3 | (3.6) | 3 | (0.9) | 0.182 |
| Cerebrovascular disease | 14 | (16.7) | 51 | (15.2) | 0.041 |
| Ischemic heart disease | 4 | (4.8) | 6 | (1.8) | 0.168 |
| Heart failure | 16 | (19.0) | 27 | (8.0) | 0.326 |
| Charson Cormorbidity Index [mean (SD)] | 5.90 | 3.71 | 4.76 | 3.44 | 0.320 |
| 0,1,2 | 14 | (16.7) | 91 | (27.1) | 0.348 |
| 3,4 | 23 | (27.4) | 112 | (33.3) |  |
| 5+ | 47 | (56.0) | 133 | (39.6) |  |
| **Smoking** (*n,* %) |  |  |  |  |  |
| No response | 0 | (0.0) | 1 | (0.3) |  |
| Never smoker | 11 | (13.1) | 60 | (17.9) | 0.239 |
| Previous smoker | 19 | (22.6) | 95 | (28.3) |  |
| Current smoker | 27 | (32.1) | 83 | (24.7) |  |
| Not available | 27 | (32.1) | 97 | (28.9) |  |
| **Alcohol use** (*n,* %) |  |  |  |  |  |
| No response | 0 | (0.0) | 1 | (0.3) | 0.224 |
| None | 41 | (48.8) | 153 | (45.5) |  |
| Mild-to-moderate | 14 | (16.7) | 64 | (19.0) |  |
| Heavy | 2 | (2.4) | 21 | (6.3) |  |
| Not available | 27 | (32.1) | 97 | (28.9) |  |
| **Cancer surgery** (*n,* %) | 17 | (20.2) | 79 | (23.5) | 0.079 |
| **Chemotherapy** (*n,* %) | 75 | (89.3) | 309 | (92.0) | 0.092 |
